# Development and psychometric properties of a Calcium Intake Questionnaire based on the social cognitive theory (CIQ-SCT) for Iranian women

**DOI:** 10.15171/hpp.2018.07

**Published:** 2018-01-07

**Authors:** Mahin Nematollahi, Ahmad Ali Eslami

**Affiliations:** ^1^Student Research Center, Isfahan University of Medical Sciences, Isfahan, Iran; ^2^Department of Health Education, Isfahan University of Medical Sciences, Isfahan, Iran

**Keywords:** Calcium intake, Social cognitive theory, Questionnaire, Reliability, Validity

## Abstract

**Background:** Osteoporosis is common among women which may be mostly due to the low intake of calcium. This article reports the development, cultural adaptation and psychometric properties of a Calcium Intake Questionnaire based on the social cognitive theory (CIQ-SCT)among Iranian women.

**Methods:** In 2016, this cross-sectional study was carried out among 400 younger than 50 years old women in Isfahan, Iran. After literature review, a preliminary 35-item questionnaire was developed. Then, forward-backward translation and cultural adaptation of the tool was conducted. Content Validity Index confirmed by an expert panel and Face Validity was evaluated in a pilot study. Exploratory and confirmatory factor analyses (EFA &CFA) were conducted on the calibration and validation sample, respectively. Reliability was also assessed using internal consistency test.

**Results:** After determining content and face validity, 20 items with 5 factors (self-efficacy,outcome expectations, social support and self-regulation) were obtained. Cronbach alpha for the instrument was found to be 0.901. In EFA, we identified a 4-factor model with a total variance of 72.3%. The results related to CFA (CMIN/DF=1.850, CFI =0.946, TLI=0.938, RMSEA=0.069[90% CI: 0.057-0.081]) indicated that the model was fit to the social cognitive theory. Self regulation was detected as the best predictor for calcium intake.

**Conclusion:** The CIQ-SCT showed acceptable levels of reliability and validity in explaining the calcium intake based on the constructs of social cognitive theory. Further psychometric testing is recommended in different population to approve the external validity of the instrument.

## Introduction

Osteoporosis is a disease in which the density and quality of bones are reduced and bones become weakened and fragile.^1^This disease is more common among women than men.^2^ Taking calcium-rich foods is important in preventing osteoporosis.^3^ Calcium is an essential ingredient for bone formation and every adult at the age of 19-49 years needs 1000 mg of calcium daily.^4^ In spite of the abundant evidence on the effects of calcium on bone health, research shows that calcium intake among women is lower than the recommended amount.^5-7^ Several studies have been conducted to investigate and identify the factors explaining calcium intake, and some questionnaires have also been developed.^8-16^

Wallston et al^8^ developed a questionnaire to measure individuals’ beliefs on calcium intake applying Multidimensional Health Locus Of Control (MHLC) scale. It contains 18 items with a 6-point Likert-type scaling format. This questionnaire explores the 3 constructs of internal control, the power of others and chance. In a study to assess the validity of MHLC, the validity of MHLC was reported to be modest and was recommended to be used with caution in future studies.^9^ The Osteoporosis Health Belief Scale (OHBS)is another inventory that measures the individual beliefs on calcium intake and contains 42 items.^10^ This tool includes perceived susceptibility, perceived severity, perceived barriers, perceived benefits and health motivation constructs. The number of questions in OHBS is high. The total variance explained by the constructs was 49.3% and internal correlation coefficient was 0.90. Internal consistency of the OHBS was determined by Cronbach alpha reliability with the coefficients ranging from 0.61 to 0.80.^11^ The reliability and validity of the tool have been confirmed in various studies.^11-13^ The Osteoporosis Self-Efficacy Scale (OSES) is another questionnaire developed by Kim et al^10^ that assesses the self-efficacy of a person about calcium intake and contains 21 items. Cronbach alpha for this scale was reported to be 0.93 and the regression coefficient and test-retest reliability were 0.94 and 0.90, respectively. Its reliability and validity have been confirmed in different studies.^10-13^The questionnaire of psychosocial variables of calcium intake was developed by Schmiege^6^ andcontains the constructs derived from 3 theories of Health Belief Model, protection motivation theory and theory of planned behavior. This questionnaire contains 64 items. It includes the structures of perceived susceptibility, perceived severity, perceived barriers, perceived benefits, self-efficacy, social norms, injunctive norms and behavioral intention. The number of questions in this questionnaire is also high. The reliability and validity of the questionnaire were supported through exploratory and confirmatory factor analyses. Cronbach alpha for the total questionnaire was reported to be 0.84.^6^ Glanz et al developed another questionnaire to assess the psychosocial concepts of calcium intake among 11 to 14 years old girls. This instrument contains 3 sets of constructs (psychosocial factors, attitudes and food preferences, and knowledge), 11 sub-sets and 61 items.^14^ Test-retest reliability was 0.73-0.78 (except for knowledge) and Cronbach alpha for the total instrument was more than 0.75. The number of items in the questionnaire was high and the validity of the tool has not been reported.^14^ The calcium intake behavior questionnaire consists of 3 sections: outcome expectations (12 items), self-efficacy (10 items), and eating behaviors (17 items). Cronbach alpha for these scales were ranged from 0.69 to 0.75 and the reliability was supported by test-retest method. However, the validity of the questionnaire is not reported.^15^

The abovementioned questionnaires have limitations in the dimension of the factors explaining calcium intake. The validity and reliability of some instruments have not completely been explained,^9^ and the most of the tools have not been normalized in Iran. Therefore, there is no proper and indigenous questionnaire for measuring calcium intake determinants in Iran, which urges the need for developing an appropriate tool to assess the explanatory factors of calcium intake. These factors are categorized in a general framework called “behavioral, cognitive, and social determinants”. Efforts to improve these variables through health education and health promotion interventions may lead to an increase in the level of calcium intake among different populations. Social cognitive theory is a prosperous theory with a broad approach that examines many factors related to calcium intake.^15,16^ Considering the fact that a calcium intake questionnaire with the characteristics like suitable validity and reliability, limited number of items, theoretically and indigenously approved has less been observed in Iran, the present study was conducted to develop and psychometrically test a questionnaire to explain the constructs of social-cognitive theory with the hope to explain calcium intake among adult women referring to Isfahan health centers.

## Materials and Methods

### Participants

Our aim in the present cross-sectional study was to determine the psychometric properties of a calcium intake questionnaire based on social cognitive theory in the city of Isfahan, from May to September 2016. In the psychometric studies, the sample size should be considered up to 15 samples per item.^17,18^ Considering the number of items in the first draft of the questionnaire 400 samples were recruited among women younger than 50 in Isfahan, Iran.

The sampling procedure was multi stage cluster random: urban health centers were randomly selected from urban and rural centers covered by Isfahan Health Center. Then, ten urban health centers were selected through cluster sampling among 25 urban centers. The population of the women covered by the centers was determined and the samples were elected in proportion to size of each center. The respondents were invited through phone calls to the centers. After giving explanations about the research objectives and obtaining informed consent, they were requested to complete the self-report questionnaires.

The inclusion criteria were completing the informed consent, and ability to response to the items. The exclusion criteria included physical and mental disability and unwillingness to complete the questionnaires.

### Instrumentation

After an extensive review on literature, a number of questionnaires were gathered.^2,6,15,16^ The questionnaires were firstly translated into Persian and the necessary reforms in terms of cultural and linguistic adaptations were conducted. All the related items were extracted to constitute an item pool (Appendix 1). Finally, the first draft of the instrument based on the constructs of social cognitive theory and calcium intake behavior was designed by a panel of experts. The questionnaire contained the following 3 components:

1. An instrument to assess 10 personal and demographic characteristics including age (year), education (illiterate/the ability to read and write/primary school/middle and high school/diploma/collegiate), marital status (married/single/widow/divorced), employment status (employed/un-employed), and income (little/moderate/good/excellent).
2. CIQ-SCT: The SCT-based questionnaire was designed by the researchers and contained 4 constructs: self-efficacy, outcome expectations, social support and self-regulation. The instrument included 35 items which were selected based on examining the relevant literature and confirmation by the research team.
3. An instrument to assess the eating behaviors about calcium-rich foods. To assess the performance of women in eating enough calcium to prevent osteoporosis, a check list similar to the food frequency questionnaire was used. The reliability and validity of this questionnaire have been confirmed in various studies.^19,20^ In this instrument, a list of calcium-rich foods was given to individuals^21^ and they were asked to determine the amount of food consumption per week and the amount of consumption per meal.^16^ Then, according to the amount of calcium contained in each food in terms of milligrams, the average daily intake of calcium is calculated and the individuals were classified into the following 3 groups: Unacceptable: the average calcium intake is less than 650 mg/d. Almost acceptable: the average calcium intake ranged from 650-1300 mg/d. Acceptable: the average calcium intake is more than 1300 mg/d.^22^

### CIQ-SCT development and validation

The reliability and validity of the questionnaire were conducted in the following 2 steps:

In order to determine the linguistic and cultural adaptation of the questionnaire, the process of translation and back-translation was conducted based on the Beaton pattern^23^ and the questionnaires were evaluated in a committee consisting of 5 bilingual experts. Two experts translated the questionnaires into Persian, and the necessary amendments were performed in accordance to the principles of translation and cultural adaptation. Then, 3 experts translated back the questionnaires into English. The final questionnaire in the research committee was reviewed and approved in terms of conforming to the original questionnaire. To determine the content validity, the developed items were examined in a 20-member panel of experts in the fields of health education and behavioral sciences. To determine CVR, the experts were asked to announce their opinion on each item in the form of a 3-part spectrum: “item is necessary”, “useful but not necessary” and “unnecessary”. According to the Lawshe table and the number of specialists participated,^20^ the CVR approval criterion for each item was considered equal to 0.42 and higher.^24^ In order to determine CVI, the opinion of experts was evaluated in 3 criteria: “simplicity”, “relevant or specific”, and “transparency and clarity”. The acceptable criterion for CVI was considered to be 0.79.^23^ In order to determine face validity (FV), the questionnaires were given to 20 women in the target group. The impact score was calculated and the scores higher than 1.5 were considered as the acceptance criteria for each item. The impact score of all items was above 1.5.^23^ At this stage, 20 items were accepted. The 4-section instrument was based on social cognitive theory constructs and the calcium intake measurement. Each scale included 5 items. The score range was based on a 10-point Likert-type scaling (1 “strongly disagree” to 10 “strongly agree”). The lowest score was 20 and the highest score was 200. The higher scores indicated the better social cognitive variables for calcium intake.

Then, the questionnaire was evaluated in a cross-sectional study in a sample of 400 women. Indicators of corrected item total correlations (CITC) included standard deviation, skewness, floor and ceiling effects were calculated, then, variance of factors, total variance, normality, internal consistency, Exploratory factor analysis (EFA), confirmatory factor analysis (CFA) and temporal stability were used to evaluate reliability of the questionnaire. Test-retest was performed in a 2-week period to confirm the repeatability of the questionnaire in a group of 30 women who were excluded from the study population. To assess the associations between the factors, correlation coefficients were computed.

### Data analysis

Data were analyzed using statistical SPSS 20 (Armonk, NY, USA) and Amos Graphic 23 (Chicago, JL, USA) software.

The samples (n = 360) were randomly divided into 2 equal groups in terms of size and basic characteristics (e.g., age, education, and income). EFA was carried out on the first sample (n_1_ = 180) and CFA was carried out on the second sample (n_2_ = 180). The first sample (calibration sample) was used to determine validity and adjust the factor structure, and the second sample (validation sample) was used to examine the stability of the developed factor structure.^25,26^ EFA was evaluated using principal component analysis (PCA) to extract the factors and Varimax method to rotate the factors. Factors were extracted using principal component (PC) method and were rotated using Varimax method. We also used Kaiser-Meyer-Olkin (KMO) measure and Bartlett test to evaluate the model adequacy. The best structure were considered to be the one with the eigenvalues greater than 1 and factor loading equal to or greater than 0.4. According to eigenvalues = 1, some items were not placed on the expected factors, so by changing the eigenvalues from 1 to 1.1, the items were aggregated into the factors well according to the expected default. In this study, the internal consistency and split-half method were used in order to examine the reliability of the scale.^27,28^

The CFA model with the robust maximum likelihood was used to estimate model parameter. The absolute fit of the model to the data was evaluated using the χ2 statistic, comparative fit index (CFI), Tucker–Lewis index (TLI), root mean square error of approximation (RMSEA). The model was considered acceptable if χ2 was between 1 and 5, CFI was greater than 0.8, TLI was more than 0.9, RMSEA was <0.05 good fit or between 0.05 and 0.08 adequate fit.^27,28^

## Results

### Descriptive statistics

Twenty-five out of 400 completed questionnaires were not included in data analysis due to lack of inclusion criteria and 15 questionnaires were deleted as a result of missing data, and finally 360 questionnaires were analyzed.

The main features of the participants included the educational level (1.4% illiterate, 3.6% the ability to read and write, 10% primary school, 13.6% middle and high school, 40.3% diploma and 31.1% collegiate), marital status (87.2% marriage, 10% single, 1.7% widow and 1.1% divorced), employment (18.9% employed, 81.1% un-employed) and income (10.6% little, 56.9% moderate, 27.8% good and 4.7% excellent). The mean age of the participants was 33.6 years (SD = 8.35), (range = 14-50). The results of eating calcium-rich foods are shown in Table 1.

### Cultural adoption

The developed questionnaire (35 items) was approved by bilingual experts and the research team. At the stage of examining CVI and CVR, 15 items were deleted: 4 items from each structure (outcome expectations, social support and self-regulation) and 3 items from the structure of self-efficacy. Also, 10 items were revised: 4 items from self-efficacy, 3 items from self-regulation, 2 items from social support and 1 item from outcome expectation. Finally, 20 items were accepted. At the stage of examining FV, 20 items with impact score higher than 1.5 were accepted. The total scale CVIs for each construct of the questionnaire were as follow: 0.73 for self-efficacy, 0.74 for outcome expectation, and 0.86 for social support. The total scale CVRs for each construct of the questionnaire were as follow: 0.8 for self-regulation, 0.75 for self-efficacy, 0.7 for outcome expectation, 0.7 for social support, and 0.81 for self- regulation. The total scale FVs for self-efficacy, outcome expectation, social support, and self-regulation were 3.82, 3.58, 3.54 and 3.86, respectively.

### Classical item analysis

Ceiling and floor effects were observed in none of the items. Table 2 shows the mean scores and standard deviations for the indicators. As the correlation coefficients of the items were higher than 0.3 and the skewness of the items were less than 1.96^29^no item was deleted (Table 2).

### Exploratory factor analysis

KMO index value was equal to 0.902, indicating the model adequacy and Bartlett’s sphericity tests results were (χ^2^ = 5090.932, *df* = 190, *P *< 0.001) which indicated the appropriateness of factor analysis on the data. EFA was performed on the calibration sample (n_1_ = 180) with Varimax method, cutoff point = 0.4 and eigenvalues = 1.1 and a 4-factor model with total variance of 72.32% was detected. The first factor was outcome expectations (items 6 to 10), the second factor was self-efficacy (items 1 to 5), the third factor was social support (items 11 to 15) and the fourth factor was self-regulation (items 16 to 20) Appendix 2. The results confirm the supposed model (Table 3).

### Reliability

Cronbach alpha of the total instrument was 0.901 that reflects inter­nal consistency as satisfactory. The internal consistency of all the factors was also good and ranged from 0.79 to 0.94 (Table 3). The results of test–retest reliability using the method of ICC in a sample of 30 women at 2 weeks intervals showed that the moderate temporal stability of the instrument. The highest amount of ICC was related to the outcome expectations (0.53; 95% CI: 0.28–0.72) and the lowest amount was related to self-efficacy (0.23; 95% CI: 0.13–0.54)). Also, no item was deleted. Spearman correlations between self-efficacy, outcome expectation, social support, self-regulation and total calcium intake use are illustrated in Table 4.

### Confirmatory factor analysis

The results showed that the measurement model had a good fit to the data in the assumed model, and all the scales were significant within an acceptable range (χ2 [166] = 307.108, P<0.001, CFI = 0.946, NFI = 0.891, TLI = 0.938, RMSEA = 0.069 [(0.57-0.081]). CFA results showed that self-regulation was the most important and the best factor in predicting calcium intake (R^2^ = 1) (Table 5).

## Discussion

Questionnaires are important tools in explaining health-related behaviors that are particularly important in the science of health education.^30,31^ The developed questionnaires based on SCT to explain the factors associated with calcium intake are very limited. The results of our study showed CIQ-SCT with acceptable validity and reliability. The developed questionnaire is based on the social cognitive theory with the most important structures of the theory. In some previously developed questionnaires, a lower number of structures from a theory have been examined.^14^ In this study, we tried to completely explain the validity and reliability stages of the questionnaire. In some previous studies, however, these stages have not been completely explained.^9^ A basic necessity for a questionnaire is the cultural and linguistic conformity with the target population. Accordingly, the developed questionnaire in our study was assessed and confirmed in terms of accuracy in translation and cultural adaptation in a panel of 5 bilingual experts. This issue has not been considered or mentioned in some other questionnaires.^14^ In order to determine the scientific validity of the questionnaire, CVI and CVR should be examined. The CVI and CVR of CIQ-SCT were conducted by a committee consisting 20 experts in the field of health education and behavioral sciences. The proper face validity of a questionnaire may lead to motivation in the respondent to answer the items. The impact score for all the items in the questionnaire was more than 1.5, which indicated the appropriateness of the items for the next analyses.

The findings of Hsieh et al^16^(GFI = 0.98, AGFI = 0.95, NFI=0.96, CFI = 0.99) and Ievers-Landis et al^32^(χ^2^ = 15.467, *df* = 18, CFI = 1.00, TLI = 1.022, RMSEA <0.001) showed the SCT as a good model to explain the behaviors of calcium intake. Our results of EFA and CFA also showed the construct validity of the questionnaire and all the scales were good indicators for their theoretical construct which confirms the results of those reported in previous studies.^13,15,16,32^

The Cronbach alpha of the entire questionnaire was 0.901 (0.795-0.943) which indicated the internal consistency reliability of the questionnaire,^33^ These findings are consistent with those that considered the Cronbach alpha to assess the reliability of the questionnaire.^16^ In this study, the Cronbach alpha for all the constructs was higher than 0.7, which was similar to those reported by Hsieh et al^16^(Cronbach alpha for self-efficacy = 0.95), Ievers-Landis et al^32^ (Cronbach alpha for self-efficacy = 0.66) and Kim and Kim^15^(Cronbach alpha for self-efficacy = 0.75 and for outcome expectations = 0.69). Also, the results of test–retest and Spearman-Brown correlation coefficients between the SCT constructs and calcium intake showed the present questionnaire with a good level of test-retest reliability indicating the instrument as a reliable tool for measuring the psychological factors associated with calcium intake among women.^11^

### Generalizability (External validity)

According to the characteristics of the target group in this study and the suitability of validity and reliability of the CIQ-SCT, it may be useful for similar populations and target groups. Further studies are suggested on the generalizability of the CIQ-SCT for other social groups and in other environments (such as hospitals and healthcare centers).

## Conclusion

The CIQ-SCT developed and validated in the present study showed acceptable levels of reliability and validity in explaining the calcium intake based on the constructs of social cognitive theory. This success may be due to the cultural and linguistic adaptation conducted in the study as well as the use of an ecological approach in investigating the individual, behavioral, and environmental factors related to calcium intake. Further psychometric testing is recommended in different population to approve the external validity of the instrument.

## Limitations

As a limitation for our study the responding format of the items in the form of self-report may be noted. The high number of items in the questionnaire may be considered as another barrier for the respondents to answer all the items. Such limitations may lead to bias in the results and, therefore, similar studies using this questionnaire may promote our understanding on the appropriateness of the instrument.

## Ethical approval

This study was conducted after gaining formal license from the Isfahan University of Medical Sciences and gaining ethical approval from Research Deputy of Isfahan University of Medical Sciences (grant No. 395203). The purpose and methodology were explained to the participants and the researcher paid attention to gain informed consent and keep the confidentiality of information

## Competing interests

The authors declare that there is no conflict of interest.

## Authors’ contributions

AAE developed the original idea, statisti­cal analysis, and manuscript revision. MN collected the data, analyzed them, and wrote the manuscript.

## Acknowledgments

This study was financially supported by Student Research Committee, School of Health, Isfahan University of Medical Sciences, Isfahan, Iran (IR.MUI.REC.1395.3.203).

We thank those who helped us in conducting this investigation, including all health department officials, health centers workers and women participated in the study.

**Table 1:** The level of calcium intake among women participated in the study (n = 360)

**Daily calcium intake**	**No.**	**%**
Not acceptable( less than 650 mg/d)	33	9.2
Almost acceptable (650-1300 mg/d)	307	85.3
Acceptable (more than 1300 mg/d)	20	5.6

**Table 2:** Classical item analysis results of the calcium intake questionnaire among women participated in the study (n = 360)

**Subject No.** ^a^	**Corrected item-total correlation**	**Squared multiple correlation**	**α If the item** **was deleted**
se1	0.594	0.718	0.895
se2	0.643	0.740	0.893
se3	0.577	0.700	0.895
se4	0.567	0.664	0.895
se5	0.544	0.676	0.896
oe1	0.535	0.757	0.896
oe2	0.557	0.737	0.895
oe3	0.513	0.720	0.897
oe4	0.523	0.767	0.896
oe5	0.524	0.789	0.896
ss1	0.462	0.646	0.898
ss2	0.549	0.648	0.896
ss3	0.566	0.732	0.895
ss4	0.446	0.518	0.899
ss5	0.478	0.530	0.898
sr1	0.468	0.414	0.898
sr2	0.433	0.349	0.899
sr3	0.540	0.429	0.896
sr4	0.532	0.451	0.896
sr5	0.595	0.718	0.894

^a^se1–se5, Self-efficacy; oe1–oe5, outcome expectations; ss1–ss5, social support; sr1–sr5, self–regulation.

**Table 3:** Exploratory factor analysis, internal consistency and the total percentage of variance for items of the questionnaire

**Items No.**	**Subject No.** ^a^	**Outcome expectation**	**Self-efficacy**	**Social support**	**Self-regulation**
1	se1	**-** ^b^	0.860	**-**	**-**
2	se2	-	0.843	-	-
3	se3	-	0.851	-	-
4	se4	-	0.846	-	-
5	se5	-	0.844	-	-
6	oe1	0.897	-	-	-
7	oe2	0.867	-	-	-
8	oe3	0.857	-	-	-
e4	0.896	-	-	-
10	oe5	0.905	-	-	-
11	ss1	-	-	0.854	-
12	ss2	-	-	0.827	-
13	ss3	-	-	0.868	-
14	ss4	-	-	0.753	-
15	ss5	-	-	0.768	-
16	sr1	-	-	-	0.711
17	sr2	-	-	-	0.689
18	sr3	-	-	-	0.630
19	sr4	-	-	-	0.668
20	sr5	-	-	-	0.733
Percentage of variance		20.822	19.760	18.025	13.721
Cronbach’s alpha		0.943	0.930	0.893	0.795

^a^ se1–se 5, Self-efficacy; oe1–oe5, outcome expectations; ss1–ss5, social support; sr1–sr5, self–regulation. ^b^ not applicable (less than 0.3).

**Table 4:** Spearman’s correlation coefficients between with the factors and calcium intake among women participated in the study (n = 360)

**Structures**	**1**	**2**	**3**	**4**	**5**
1: Self-efficacy	0.251	0.475	0.234	0.321	1
2: Outcome expectation	0.112	0.280	0.194	1	0.321
3: Social support	0.176	0.469	1	0.194	0.234
4: Self-regulation	0.270	1	0.469	0.280	0.475
5: Total calcium intake	1	.270	0.176	0.112	0.251

Correlation is significant at the 0.01 level (two-tailed).

**Appendix 1.** Items of the initial questionnaire

**Subject No.** ^a^	**Initial questionnaire items**	
se1	I can change my diet plan to increase the consumption of calcium intake	-
se2	I can eat high calcium foods	Deleted
se3	I can remember to eat calcium supplements daily	Deleted
se4	I can prefer eating dairy products to snacks	-
se5	I can use buttermilk and milk instead of coffee and beverages	-
se6	I can use calcium-enriched foods if I go out with my friends.	-
se7	I cannot buy expensive calcium materials	-
se8	I can use chalcedony when traveling	Deleted
oe1	Calcium intake reduces the risk of osteoporosis	-
oe2	Dairy products (e.g., milk & cheese) are tastier than snacks (e.g., snacks & chips)	-
oe3	Dough and milk are as sweet and delicious as fruits	Deleted
oe4	Eating dairy products (e.g., milk, buttermilk, & yogurt) cause indigestion and abdominal pain	-
oe5	Dough and milk are not tastier than soda and coffee	-
oe6	Green leafy vegetables (broccoli-cabbage) are dreadful	-
oe7	Dairy and vegetables should not be eaten because they are raw	Deleted
oe8	Dairy and vegetables are more nutritious than other foods	Deleted
oe9	Using calcium supplements is difficult because they are expensive	Deleted
ss1	My family and friends talk about the use of calcium bromine	Deleted
ss2	My family and friends are worried about the lack of food and the consumption of calcium-rich foods (e.g., milk & yogurt)	-
ss3	My family and friends have positive comments about calcium intake	Deleted
ss4	My family and friends encourage me to eat calcium-rich foods (such as milk and buttermilk)	-
ss5	My family uses calcium carbonate foods when eating meals	Deleted
ss6	My family has at least one meal of dairy or fish per day	-
ss7	My family uses calcium-based foods in family visits	‏-
ss8	My family uses dough instead of soft drinks and coffee	Deleted
ss9	My family eats dairy products (e.g., milk, yogurt) at least twice a day	‏-
sr1	I have a certain diet to use calcium-rich foods	‏-
sr2	I plan the use of calcium-rich foods according to the family income	‏-
sr3	In the diet, I use dairy products, vegetables and fish	Deleted
sr4	I am persistent and delighted in the implementation of a calcium nutrition program	Deleted
sr5	I use calcium-containing substances in the diet plan	Deleted
sra6	Using calcium-rich foods is the important priority of my nutrition.	‏-
sr7	I have written a nutritionally calcium diet plan	Deleted
sr8	I use high calcium and affordable foods in the diet plan	‏-
sr9	‏In the preparation of breakfast, I use a variety of materials with calcium, such as milk, cheese, walnuts and almonds	‏-

^a^ se1-se 8, Self-efficacy; oe1–oe9, outcome expectations; ss1–ss9, social support; sr1– sr9, self–regulation.

**Table 5:** Indices of first-order and second-order confirmatory models (n = 180)

**Models**	**CMIN**	**DF**	**CMIN/DF**	**NFI**	**CFI**	**TLI**	**RMSEA (LO-HI)**
First order model	298.856	164	1.822	0.894	0.949	0.940	0.068 (0.055-0.080)
Second order model	307.108	166	1.850	0.891	0.946	0.938	0.069 (0.057-0.081)

χ^2^ values are significant (P < 0.005).

**Appendix 2.** Final questionnaire

**Item No.**	**Factors** ^a^	**Items**
1	se1	In my daily diet, I can use calcium-rich foods such as milk, cheese and yogurt.
2	se2	At leisure time, I can use dairy products (milk & cheese) rather than junk food (snacks & chips).
3	se3	I can use buttermilk and milk instead of coffee and beverage.
4	se4	Even when sightseeing with my friends, I can use calcium-rich foods such as walnuts and almonds.
5	se5	Despite the high cost of some calcium-rich foods, such as cheese, walnuts, almonds and pistachios, I can prepare them.
6	oe1	Calcium-rich foods such as milk and cheese reduce the risk of osteoporosis.
7	oe2	Dairy products (e.g., milk & cheese) are tastier than snacks (e.g., snacks & chips).
8	oe3	Eating dairy products (e.g., milk, buttermilk, & yogurt) cause indigestion and abdominal pain.
9	oe4	Buttermilk and milk are tastier than coffee and beverage.
10	oe5	Green leafy vegetables (e.g., broccoli, kale) cannot be eaten because of being raw.
11	ss1	My family and friends are worried about the lack of food and the consumption of calcium-rich foods (e.g., milk & yogurt).
12	ss2	My family eats dairy products (e.g., milk, yogurt) at least twice a day.
13	ss3	My family and friends encourage me to eat calcium-rich foods (such as milk and buttermilk).
14	ss4	My family dedicate at least one meal a week to eat fish.
15	ss5	My family and friends use calcium-rich foods (e.g., milk, yogurt & cheese) instead of snacks (e.g., beverages, snacks & chips) at the leisure time.
16	sr1	I have a certain diet to use calcium-rich foods.
17	sr2	I plan to use calcium-rich foods according to the family income.
18	sr3	In the preparation of breakfast, I use a variety of materials with calcium, such as milk, cheese, walnuts and almonds.
19	sr4	At daily shopping, I buy foods which have higher calcium and are less expensive.
20	sr5	Using calcium-rich foods is an important priority in my nutrition.

^a^ se1-se 8, Self-efficacy; oe1–oe9, outcome expectations; ss1–ss9, social support; sr1– sr9, self–regulation.
